# Cause rare d’hémorragie digestive haute: rupture d’un faux anévrisme de l’artère gastroduodénale

**DOI:** 10.11604/pamj.2021.38.208.28207

**Published:** 2021-02-24

**Authors:** Issam Loukil, Amine Zouari

**Affiliations:** 1 Service de Chirurgie Générale Tataouine, Tataouine, Tunisie,; 2 Service de Chirurgie Générale Sfax, Sfax, Tunisie

**Keywords:** Anévrisme, artère, gastroduodénale, Aneurysm, artery, gastroduodenal

## Abstract

A 69-year-old female patient with diabetes and hypertension on treatment presented in the emergency department with haematemesis. She reported intermittent epigastric pain evolving for a few days. Clinical examination only showed epigastric susceptibility. Laboratori tests revealed severe normocytic normochromic anemia (Hb=8.3 gr/dl), normal amylasemia with preservation of liver function and coagulation. Patient´s outcome was marked by decline in haemoglobin concentrations and haemodynamic instability despite good venous filling and blood transfusion. Given the nonavailability of urgent digestive endoscopy, abdominal angioscan showed gastroduodenal artery pseudoaneurysm measuring 6mm along its longer axis without signs of active leakage or intraperitoneal effusion, associated with choledocoduodenal fistula. The diagnosis of heavy upper gastrointestinal bleeding secondary to gastroduodenal artery pseudoaneurysm rupture was retained. The patient underwent emergency surgery given the nonavailability of arterial embolization through vascular ligation. Patient´s outcome was favorable with stabilization of haemoglobin level and hemodynamic status.

## Image en médecine

Une patiente âgée de 69 ans, diabétique et hypertendue sous traitement, consulte aux urgences pour des hématémèses. Elle rapporte des douleurs épigastriques intermittentes évoluant depuis quelques jours. L´examen clinique trouve seulement une sensibilité épigastrique. La biologie a révélé une anémie sévère (Hb=8.3 gr/dl) normochrome normocytaire, une amylasémie normale, avec conservation de la fonction hépatique et de la coagulation. L´évolution a été marquée par la chute du taux d´hémoglobine et altération de l´état hémodynamique malgré un bon remplissage vasculaire et transfusion sanguine. Devant la non disponibilité d´une endoscopie digestive immédiate, un angioscanner abdominal a décrit l´image d´un faux anévrysme de l´artère gastroduodénale de 6mm de grand axe sans signes de fuite active ni d´épanchement intrapéritonéal, associée à un aspect de fistule cholédocoduodénale. On a retenu le diagnostic d´une hémorragie digestive haute de grande abondance secondaire à une rupture d´un faux anévrysme de l´artère gastroduodénale. La patiente a été opérée en urgence, devant le non disponibilité d´un geste d´embolisation artérielle, par une ligature vasculaire. L´évolution a été favorable avec stabilisation des chiffres d´hémoglobine et de l´état hémodynamique.

**Figure 1:**
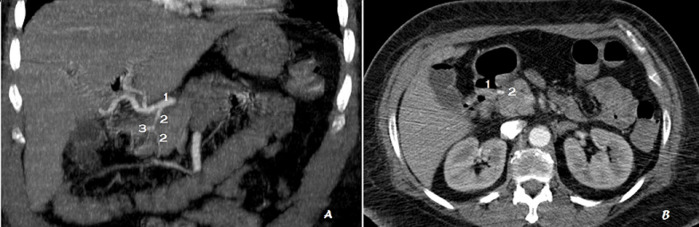
A) angio-tomodensitométrie (TDM) abdominale (reconstruction coronale oblique) anévrisme sacciforme de l'artère gastroduodénale; 1: artère hépatique commune; 2: artère gastroduodénale; 3: anévrisme sacciforme; B) TDM abdominale en coupe axiale anévrisme sacciforme de l'artère gastroduodénale qui déborde dans la lumière duodénale; 1: lumière duodénale; 2: anévrisme sacciforme de l'artère gastroduodénale

